# Downregulation of TFAM inhibits the tumorigenesis of non-small cell lung cancer by activating ROS-mediated JNK/p38MAPK signaling and reducing cellular bioenergetics

**DOI:** 10.18632/oncotarget.7018

**Published:** 2016-01-25

**Authors:** Deyao Xie, Xiaoyi Wu, Linhua Lan, Fugeng Shangguan, Xiaoming Lin, Fuhong Chen, Shan Xu, Ya Zhang, Zilei Chen, Kate Huang, Rongrong Wang, Lu Wang, Xiaoxiao Song, Yongzhang Liu, Bin Lu

**Affiliations:** ^1^ Department of Cardiothoracic Surgery, The First Affiliated Hospital of Wenzhou Medical University, Wenzhou, Zhejiang 325035, P.R. China; ^2^ Protein Quality Control and Diseases Laboratory, Attardi Institute of Mitochondrial Biomedicine, School of Life Sciences, Wenzhou Medical University, Wenzhou, Zhejiang 325035, P.R. China; ^3^ Huzhou Health School, Huzhou, Zhejiang 313100, P.R. China; ^4^ Department of Pathology, The First Affiliated Hospital of Wenzhou Medical University, Wenzhou, Zhejiang 325035, P.R. China; ^5^ Department of Otolaryngology, The First Affiliated Hospital of Wenzhou Medical University, Wenzhou, Zhejiang 325035, P.R. China

**Keywords:** mitochondria, TFAM, non-small cell lung cancer, chemosensitivity, cellular bioenergetics

## Abstract

Mitochondrial transcription factor A (TFAM) is essential for the replication, transcription and maintenance of mitochondrial DNA (mtDNA). The role of TFAM in non-small cell lung cancer (NSCLC) remains largely unknown. Herein, we report that downregulation of TFAM in NSCLC cells resulted in cell cycle arrest at G1 phase and significantly blocked NSCLC cell growth and migration through the activation of reactive oxygen species (ROS)-induced c-Jun amino-terminal kinase(JNK)/p38 MAPK signaling and decreased cellular bioenergetics. We further found that TFAM downregulation in NSCLC cells led to increased apoptotic cell death and enhanced the sensitivity of NSCLC cells to cisplatin. Tissue microarray (TMA) data showed that elevated expression of TFAM was related to the histological grade and TNM stage of NSCLC patients. We also demonstrated that TFAM is an independent prognostic factor for overall survival of NSCLC patients. Taken together, our findings suggest that TFAM could serve as a potential diagnostic biomarker and molecular target for the treatment of NSCLC, as well as for prediction of the effectiveness of chemotherapy.

## INTRODUCTION

Lung cancer is still the leading cause of cancer-related deaths worldwide, accounting for over 1.37 million deaths annually. There are two main types of lung cancer grouped according to cell morphology, namely small cell lung cancer (SCLC) that is the most aggressive type and accounts for about 15% of all cases and non-small cell lung cancer (NSCLC) that accounts for more than 85% of all cases [1–3]. Despite recent improvements in early diagnosis/screening and development of novel therapeutic strategies, the prognosis for all stages of NSCLC is poor: the 5-year survival for all combined stages remains less than 15% due to cancer metastasis at the time of diagnosis and relapse [1, 4–6]. Therefore, it is urgent to identify novel biomarkers for the diagnosis and better understanding of the molecular mechanism of NSCLC tumorigenesis, as well as for finding more efficient chemotherapeutic targets for developing novel treatment strategies for NSCLC.

Mitochondria play a critical role in ATP production through oxidative phosphorylation as well as in cell death or survival. TFAM is a member of the high-mobility group (HMG) protein family that is encoded by the nuclear genome and transported into the mitochondrial matrix after translation in the cytoplasm; TFAM is essential for the replication, transcription and maintenance of mtDNA [7–11].

Treatment of KB human epidermoid cancer cells and HCT 116 human colon adenocarcinoma cells with cisplatin or 5-FU leads to up-regulation of TFAM expression [12]. p53 physically interacts with TFAM to regulate cell death and it preferentially binds to cisplatin-damaged mtDNA within mitochondria [12]. The outcomes of colorectal cancer patients whose tumor tissues had high expression of TFAM were worse than those of patients who had low TFAM expression in tumor tissues [13]. However, the role and molecular mechanism of TFAM in the development and progression of NSCLC remains elusive. Therefore, a better understanding of the molecular mechanisms underlying the relationship between NSCLC tumorigenesis and TFAM expression will facilitate better approaches in cancer treatment.

To explore the role of TFAM in the tumorigenesis of human NSCLC, we analyzed the effects of TFAM knockdown on the cell cycle, cell proliferation and apoptosis in NSCLC cells, as well as on cellular bioenergetics. Moreover, we investigated the regulatory effect of TFAM depletion on the JNK/p38 MAPK signaling pathway and the sensitivity of NSCLC cells to chemotherapeutic drugs. TFAM-knockdown NSCLC H460 cells lost the ability to develop tumor masses when injected into nude mice. To further understand the clinical significance of TFAM expression in NSCLC progression, we employed tissue microarray (TMA) to examine the expression patterns of TFAM protein in a large cohort of NSCLC patients' specimens and assessed the relationship between TFAM expression and clinicopathological features of NSCLC patients. Our findings indicate that TFAM could be used as a potential clinical diagnostic and/or prognostic biomarker, as a novel therapeutic target for NSCLC, and in predicting the effectiveness of chemotherapy.

## RESULTS

### TFAM knockdown inhibits NSCLC cell growth, migration and tumor growth

**Figure 1:**
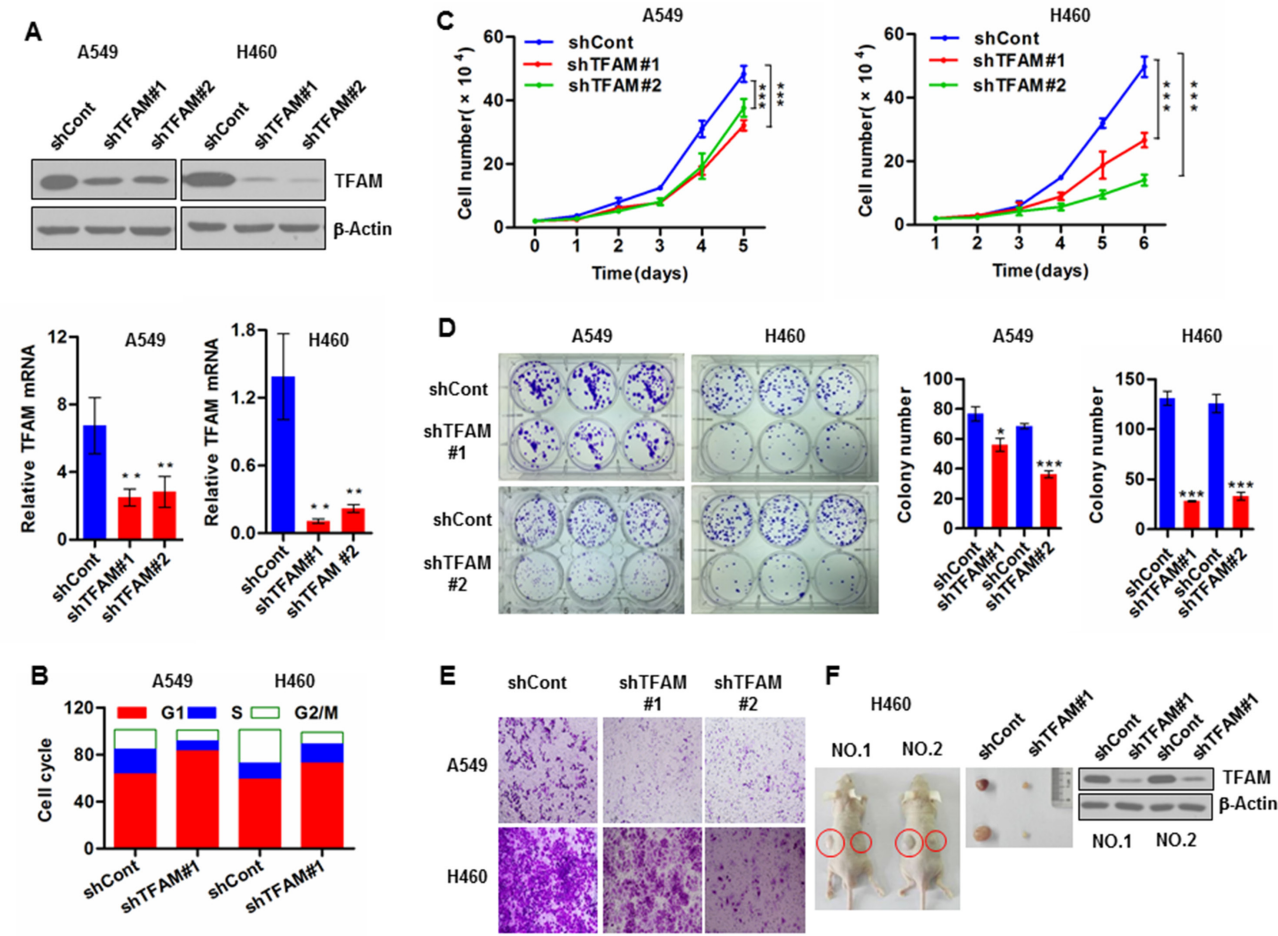
TFAM knockdown inhibits NSCLC cell proliferation and tumor growth (**A**) Upper panel: Western blot analysis demonstrating TFAM stable knockdown in the indicated NSCLC cells transfected with shRNA specific to *TFAM* mRNA. Lower panel: Real-time qPCR analysis of *TFAM* mRNA in the indicated TFAM stable knockdown NSCLC cells transfected with shRNA specific to *TFAM* mRNA. Representative data were shown from three independent experiments. Data are shown as mean ± SD (*n* = 3, **p < 0.01). (**B**) At 48 hr after incubation at 37°C in a CO_2_ incubator, TFAM stable knockdown and vector control NSCLC cells were harvested and stained with PI (50 μg/ml) solution. Cell cycle arrest was analyzed with a BD Accuri^™^ C6 flow cytometer. Results are representative of three independent experiments. (**C**) Cell proliferation of stable TFAM knockdown NSCLC A549 (left panel) and H460 (right panel) cells, measured by cell number counting. The data are presented as mean ± SD (*n* = 3, ***p < 0.001). (**D**) Representative images of colony formation assay of control and TFAM knockdown stable NSCLC cell lines A549 and H460 (left panel). The graphs represent the mean ± SD of at least three independent colony formation assays each performed in triplicate (middle and right panels). (**E**) Representative images of transwell migration assay of NSCLC A549 (upper panel) and H460 (lower panel) cells. Migrated cells were stained with crystal violet, photographed and counted. Data are presented as mean ± SD of at least three independent experiments. (**F**) Representative dissected tumors and TFAM expression in tumor tissue lysates are shown.

To explore the potential roles of TFAM in NSLCC tumorigenesis, we stably knocked down TFAM in NSCLC A549 cells and H460 cells with shRNA specific to TFAM mRNA. Downregulation of TFAM protein and mRNA levels was confirmed by immunoblotting and quantitative real-time PCR (qRT-PCR), respectively (Figure 1A). mtDNA copy numbers were reduced in TFAM knockdown NSCLC cells, as determined by qRT-PCR (Figure S1A). We also determined mtDNA copy numbers in NSCLC tumor tissues and matched tumor-adjacent normal tissue (Figure S1B). Knockdown of TFAM led to cell cycle arrest at G1 phase (Figure 1B), and caused inhibition of cell proliferation (Figure 1C) in both A549 and H460 cells. Similar results were obtained from a colony formation assay (Figure 1D). These results indicated that TFAM knockdown could suppress NSCLC cell growth. TFAM knockdown also led to significant reduction in cell migration as monitored in the transwell assay (Figure 1E). Moreover, we performed a xenograft experiment in which nude mice were subcutaneously injected with vector control and TFAM stable knockdown H460 cells on the left and right flanks, respectively. We found that TFAM knockdown resulted in significantly reduced tumor growth (Figure 1F).

### TFAM knockdown promotes ROS dependent activation of JNK/p38 MAPK and apoptosis

**Figure 2:**
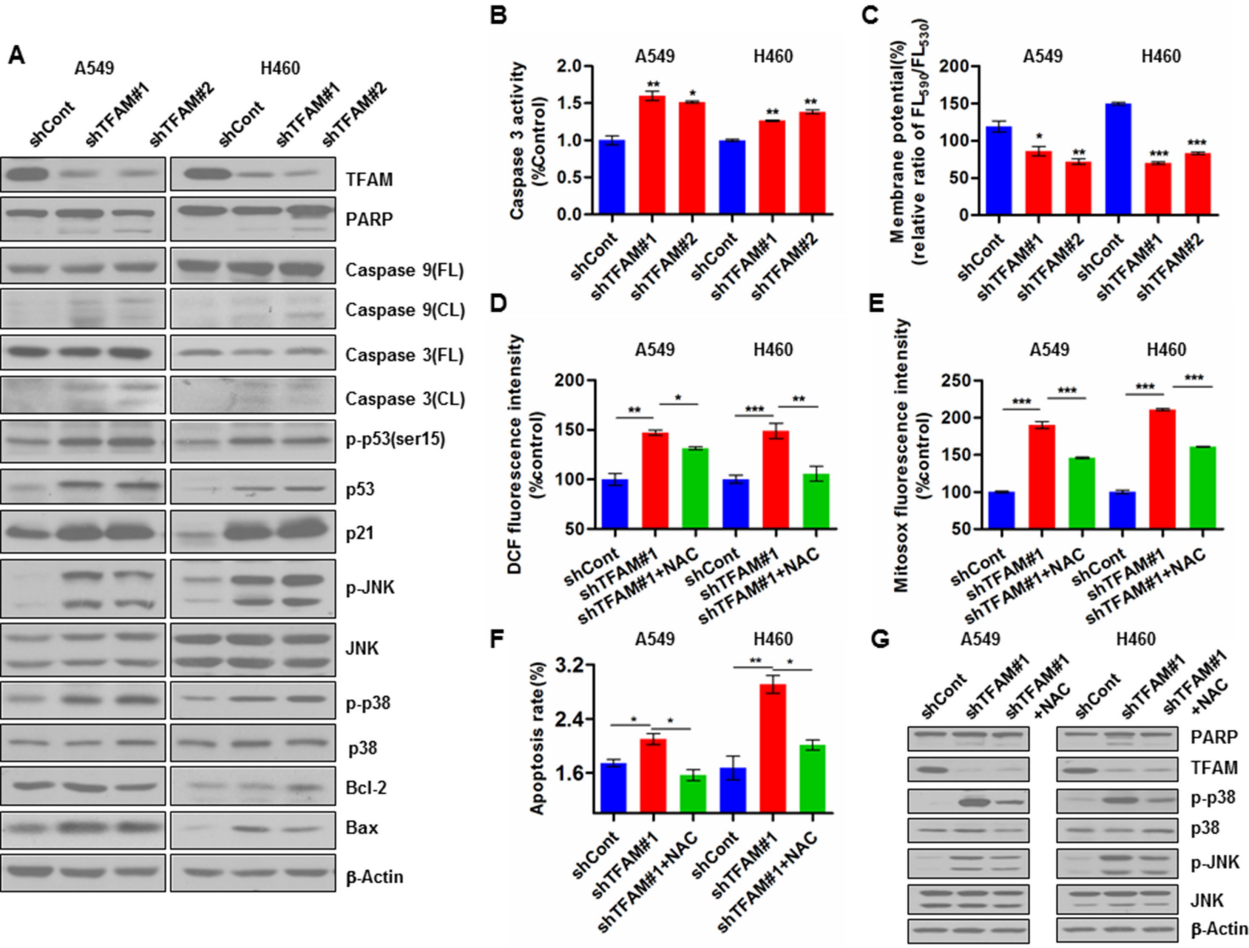
TFAM knockdown promotes ROS production and apoptosis of NSCLC cells (**A**) TFAM stable knockdown in NSCLC A549 cells (left) and H460 cells (right) increases p53, p-p53(Ser15), p21, p-JNK, Bax and p-p38 expression, as well as the cleavage of PARP, caspase 3 and caspase 9. (**B**) TFAM stable knockdown in NSCLC A549 and H460 cells increases caspase-3 activity. The data are presented as mean ± SD. *n* = 3, *p < 0.05; **p < 0.01. (**C**) TFAM stable knockdown reduces mitochondrial membrane potential (MMP) of NSCLC A549 and H460 cells. Cells were stained with JC-1 and analyzed by flow cytometry. The ratio of fluorescence intensities Ex/Em = 490/590 and 490/530 nm (FL590/FL530) were recorded to show the MMP level of each sample. Data are presented as mean ± SD. *n* = 3, *p < 0.05; **p < 0.01, ***p < 0.001. (**D**) TFAM stable knockdown increases intracellular ROS (H_2_O_2_) production in NSCLC A549 and H460 cells measured by Reactive Oxygen Species Assay Kit (DCFH-DA), and pre-treatment of cells with NAC (4 mM) for 48 hr resulted in reduction of intracellular ROS (H_2_O_2_) levels. Data are plotted as percentage of increase in median fluorescence intensity (MFI) and shown as mean ± SD (*n* = 3, *p < 0.05, **p < 0.01). (**E**) Mitochondrial superoxide levels of control and TFAM knockdown stable NSCLC A549 and H460 cells were detected by MitoSox staining and analyzed by flow cytometry. Pre-treatment of cells with NAC (4 mM) for 48 hr resulted in reduction of mitochondrial ROS production. Data are plotted as percentage of alteration in mean fluorescence intensity (MFI) and shown as mean ± SD (*n* = 3, *p < 0.05, **p < 0.01). (**F**) TFAM stable knockdown increases apoptosis rate of NSCLC A549 and H460 cells, and the pre-treatment of cells with NAC (4 mM) for 48 hr attenuates the apoptosis rate. The data shown represent results from three independent experiments (*p < 0.05; **p < 0.01). (**G**) Immunoblot detecting expression of PARP, cleaved PARP, p38, p-p38, JNK, p-JNK in lysates from control and TFAM stable knockdown NSCLC cells, as well as from cells treated with NAC (4 mM) for 48 hr. β-actin was used as loading control.

We next examined the effect of TFAM knockdown on apoptosis-related proteins in NSCLC cells. Our results showed that TFAM stable knockdown in NSCLC A549 and H460 cells led to increased expression of p53, p-p53 (ser15), p21, p-JNK, p-p38 and pro-apoptotic Bax, as well as the cleavage of PARP, caspase 3 and caspase 9; the expression of Bcl-2, which inhibits apoptosis, remained unchanged (Figure 2A). In addition, we measured caspase 3 activity, which again showed an increased activation in the TFAM knockdown NSCLC cells (Figure 2B). The caspase-dependent apoptosis rate in TFAM knockdown cells is quite stable, as shown in Figure S2.

We further determined the impact of TFAM knockdown in NSCLC cells on mitochondrial membrane potential (MMP) and ROS generation. TFAM knockdown led to dramatic MMP depolarization (Figure 2C), and significant elevation of both intracellular ROS (H_2_O_2_) and mitochondrial ROS (superoxide) levels in A549 and H460 cells, which can be scavenged upon the addition of the ROS scavenger N-acetyl-L-cysteine (NAC) (Figure 2D and 2E). Moreover, FACS assay showed that TFAM knockdown triggered significantly increased cellular apoptosis (Figure 2F), while the apoptosis rate was decreased in NAC pre-treated cells (Figure 2F).

We then examined the phosphorylation of JNK and p38 MAPKs, which may potentially be activated by ROS to induce apoptosis. Indeed, TFAM stable knockdown in both A549 and H460 cells resulted in significant increase of JNK and p38 phosphorylation levels (Figure 2G). NAC pre-treatment significantly reduced p38 phosphorylation, but surprisingly only resulted in a slight reduction in JNK phosphorylation in both TFAM knockdown NSCLC cells (Figure 2G).

### TFAM depletion enhances chemosensitivity of NSCLC cells by promoting ROS-induced caspase-dependent apoptosis

**Figure 3:**
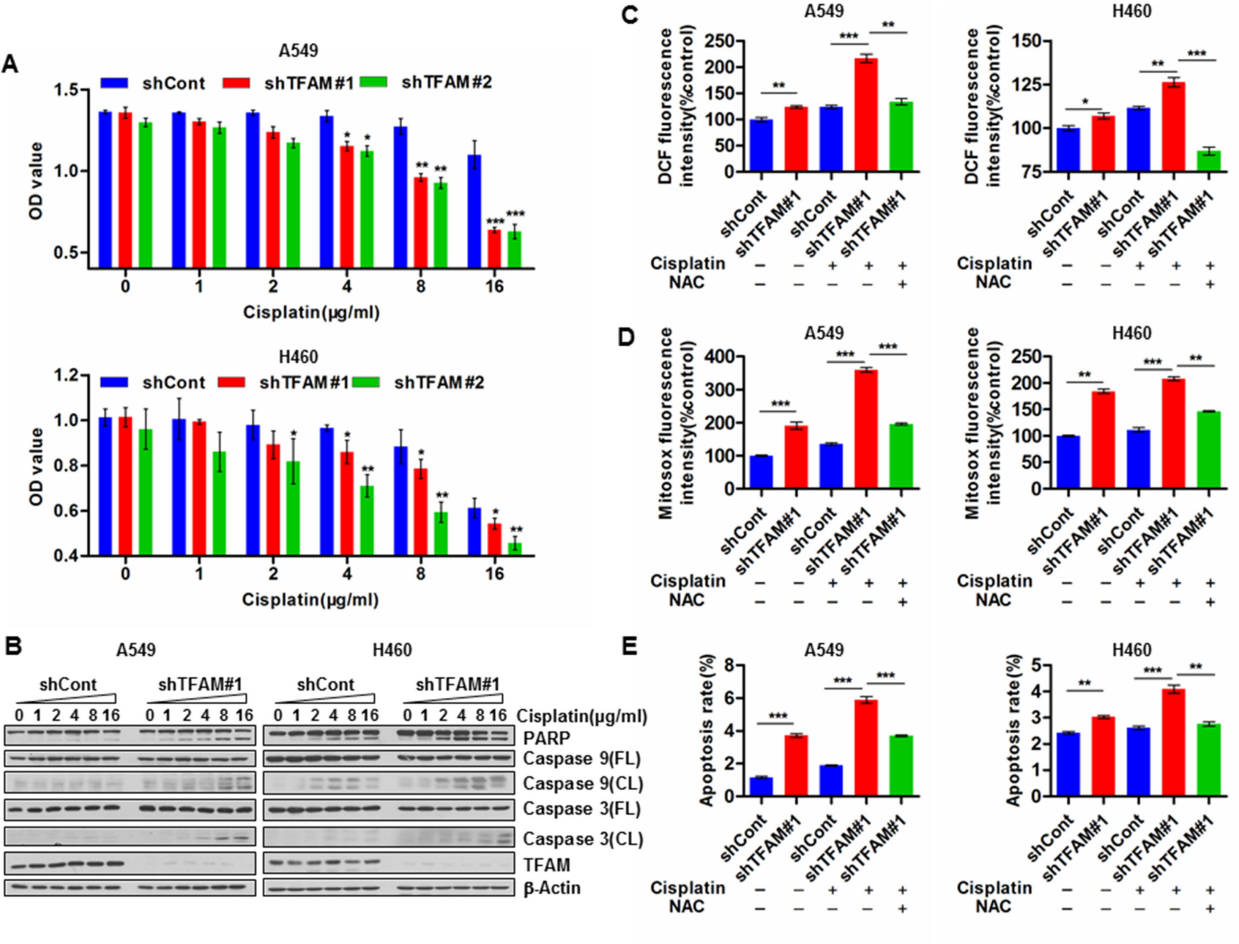
TFAM knockdown enhances chemosensitivity of NSCLC cells by facilitating ROS induced caspase-dependent apoptosis (**A**) TFAM stable knockdown NSCLC A549 (upper) and H460 (lower) cells were treated with the indicated concentration of cisplatin. At 24 hr post-cisplatin or -saline treatment, cell viability was assayed by CCK-8. The data are presented as mean ± SD (*n* = 3, *p < 0.05; **p < 0.01; ***p < 0.001). (**B**) Western blot analysis of TFAM, PARP, cleaved PARP, caspase 9, cleaved caspase 9, caspase 3 and cleaved caspase 3 in lysates from control and TFAM stable knockdown NSCLC A549 and H460 cells treated with indicated concentration of cisplatin for 24 hr. β-actin was used as loading control. (**C**) The effects of TFAM knockdown on intracellular ROS (H_2_O_2_) production induced by cisplatin (10 μg/ml) in NSCLC A549 (left) and H460 (right) cells with or without NAC (4 mM) pre-treatment for 48 hr. The intracellular ROS (H_2_O_2_) was measured by ROS assay kit (DCFH-DA). Data are presented as mean ± SD (*n* = 3, *p < 0.05; **p < 0.01; ***p < 0.001). (**D**) The effects of TFAM knockdown on mitochondrial ROS (superoxide) production induced by cisplatin (10 μg/ml) in NSCLC A549 (left) and H460 (right) cells with or without NAC (4 mM) pre-treatment for 48 hr. The mitochondrial ROS (superoxide) was detected by MitoSox staining and analyzed by flow cytometry. Data are presented as mean ± SD (*n* = 3, **p < 0.01; ***p < 0.001). (**E**) TFAM stable knockdown increases cisplatin (10 μg/ml)-induced apoptosis rate of NSCLC A549 and H460 cells with or without NAC (4 mM) pre-treatment for 48 hr. Data represent results of three independent experiments (**p < 0.01; ***p < 0.001).

We next asked whether elevated TFAM was involved in the acquisition of resistance to chemotherapeutic agents by decreasing the efficacy of cytotoxic reagents to promote NSCLC progression. To test this hypothesis, we investigated the effects of cisplatin on TFAM stable knockdown NSCLC cells or vector control cells. TFAM knockdown significantly decreased cell viability in a dose-dependent manner (Figure 3A; *n* = 3; *p < 0.05; **p < 0.01; ***p < 0.001). Moreover, TFAM-knockdown NSCLC A549 and H460 cells exhibited elevated cleavage of PARP, caspase 9 and caspase 3 compared with vector control cells (Figure 3B). Thus, TFAM depletion enhances cisplatin-induced caspase-dependent apoptosis. A potential explanation for this phenomenon could be increased cisplatin-induced ROS generation in TFAM-knockdown NSCLC cells; conversely, however, ROS levels were reduced by NAC pre-treatment (Figure 3C and 3D; *n* = 3; *p < 0.05; **p < 0.01; ***p < 0.001). Furthermore, NAC pre-treatment of TFAM stable knockdown NSCLC cells led to significant suppression of cisplatin-induced apoptosis (Figure 3E).

### TFAM knockdown inhibits mitochondrial respiration and glycolysis in NSCLC cells

**Figure 4:**
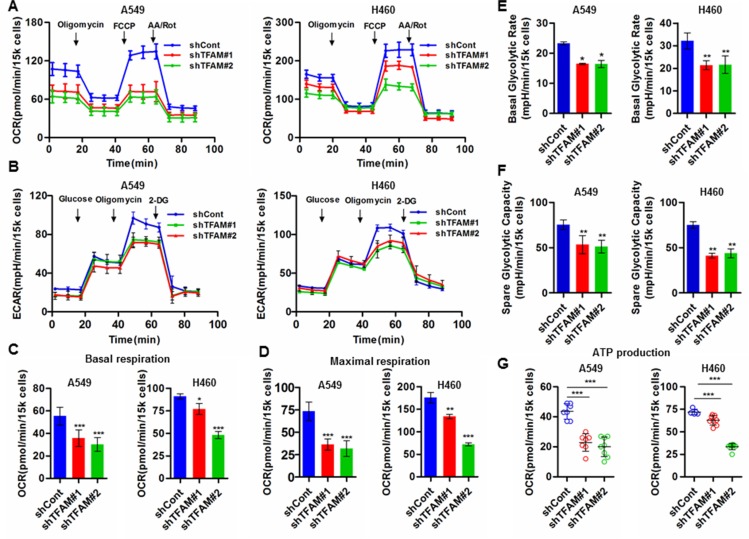
TFAM knockdown inhibits mitochondrial respiration and glycolysis in NSCLC cells (**A**) Mitochondrial respiration profile of TFAM stable knockdown NSCLC A549 and H460 cells. The intact cellular oxygen consumption rate (OCR) was measured in real time using the Seahorse XF96 Extracellular Flux Analyzer. Basal OCR were measured at three time points, followed by sequential injection of the ATP synthase inhibitor oligomycin (1 μM), the uncoupler FCCP (1 μM), the complex I inhibitor rotenone (1 μM) and complex III inhibitor antimycin A (1 μM). The representative graph represents the mean OCR ± SD of six replicates. (**B**) Extracellular acidification rate (ECAR) was detected by the Seahorse XF96 Extracellular Flux Analyzer. Injection order: Glucose (10 mM), Oligomycin (1 μM), 2-DG (100 mM). (**C** and **D**) Basal and maximal respiration (OCR) of A549 and H460 cells transfected with control and TFAM shRNA. (**E** and **F**) Basal glycolytic rate and spare glycolytic capacity were analyzed by overall ECAR in control and TFAM knockdown cells. (**G**) ATP production of control (shCont) and shTFAM groups were calculated by the OCR of baseline minus oligomycin treatment. Data are presented as mean ± SD (*n* = 6, ***p < 0.001).

TFAM plays critical roles in mtDNA replication and transcription, as well as in the maintenance of mtDNA. To investigate the effect of TFAM knockdown on the cellular bioenergetics of NSCLC cells, we analyzed the oxygen consumption rate (OCR) and extracellular acidification rate (ECAR) of living TFAM-downregulated NSCLC cells by extracellular flux analyzer. TFAM downregulation markedly decreased mitochondrial respiration in NSCLC cells (Figure 4A), and reduced glycolysis rates as indicated by ECAR in both A549 and H460 cells (Figure 4B). We further analyzed the indices that represent alteration of mitochondrial respiration and glycolysis, and found that both basal and maximal respiration were remarkably reduced in TFAM knockdown cells, which indicated some disruption of oxidative phosphorylation (OXPHOS; Figure 4C and 4D). Interestingly, our data also showed that TFAM knockdown resulted in the decrease of basal glycolytic rate and spare glycolytic rate capacity, which suggest that TFAM depletion may lead to certain retrograde signaling that communicates with the nucleus and subsequently modulates signal transduction pathways (Figure 4E and 4F). Finally, we found less ATP production in TFAM-knockdown NSCLC cells, which may not meet cellular ATP demands to support cell proliferation, migration and cell growth (Figure 4G). Although further studies need to be done to uncover the molecular mechanisms involved, our data suggest that TFAM acts as a crucial modulator of cellular bioenergetics in NSCLC cells.

### TFAM expression in TMA and its correlation with clinicopathological features of NSCLC

**Figure 5:**
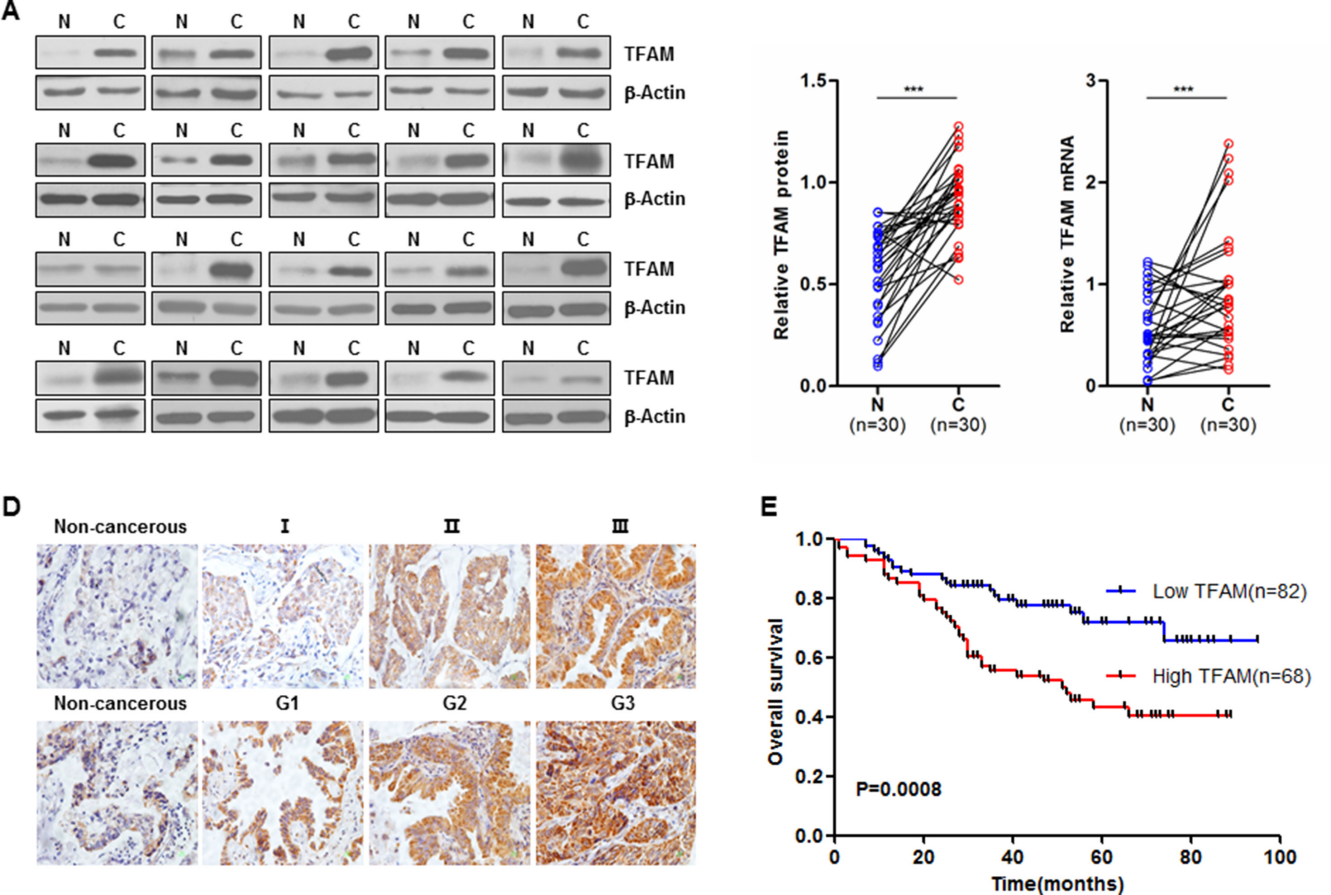
TFAM overexpression in NSCLC is closely associated with poor outcomes (**A**) Representative western blots (left panel) are shown of TFAM protein expression in NSCLC tumor adjacent normal (N) and matched tumor tissues (C) (*n* = 30). Relative TFAM protein expression of western blotting analyses (middle panel) were quantified using Image J and normalized to the β-actin internal control (*n* = 30, ***p < 0.001). TFAM mRNA in NSCLC tumor adjacent normal (N) and matched tumor tissues (**C**) were examined by qRT-PCR (right panel, *n* = 30, ***p < 0.001). Expression levels of TFAM protein and mRNA are shown as mean ± SD. (**B**) Immunohistochemical (IHC) staining of TFAM protein in NSCLC tumor tissues. Representative TFAM IHC staining photomicrographs (400 ×) of non-cancerous, Grade 1 (G1), Grade 2 (G2) and Grade 3 (G3) NSCLC tumor tissues (upper panel), and non-cancerous, TNM stage I, II and III NSCLC tumor tissues (lower panel), are shown. (**D**) Kaplan-Meier overall survival curve of NSCLC cancer patients (*n* = 150) according to TFAM protein expression (*p* = 0.0008).

To investigate whether TFAM expression is associated with tumor progression in NSCLC, western blot analysis was performed on samples from 30 NSCLC patients (each sample including tumor tissue and matched adjacent normal tissue from the same patient). TFAM protein expression was markedly increased in tumor tissues (Figure 5A and 5B; *n* = 30; ***p < 0.0001). In addition, the TFAM mRNA level was significantly increased in NSCLC tissues compared with matched adjacent normal tissues (Figure 5C; *n* = 30; ***p < 0.0001). To further test whether TFAM expression is elevated in the tumor tissues and to determine its association with clinical and pathologic parameters of NSCLC patients, we performed IHC in TMA containing 150 archived paraffin-embedded NSCLC specimens. Representative IHC images of different pathological grade and TNM stage are shown in Figure 5D.

The association between TFAM protein expression and clinicopathological features of NSCLC was analyzed by the chi-square test. As shown in Table 1, our data demonstrated that the expression of TFAM was significantly associated with TNM stage (Table 1; *p* = 0.014) and pathological grade (Table 1; *p* = 0.005). No significant relationship was found between TFAM expression and variables such as gender, age, T stage, lymph node metastases, smoking and alcohol status, however.

**Table 1:** Association between TFAM expression and various clinicopathological factors of NSCLC patients

Variables	No. (*n* = 150)	TFAM protein expression		*p* value
		Low (*n* = 82)	High (*n* = 68)	
**Gender**				
Male		52	48	0.354
Female		30	20	
**Alcohol**				
Yes		22	18	0.961
No		60	50	
**Age**				
< 61		37	31	0.954
≥ 61		45	37	
**Smoking history**				
Yes		40	38	0.386
No		42	30	
**Grade**				
G1		12	4	**0.005**
G2		55	36	
G3		15	28	
**T stage**				
T1		9	2	0.152
T2		67	59	
T3–T4		6	7	
**Lymph node metastases (N)**				
N 0		50	39	0.653
N ≥ 1		32	29	
**TNM stage**				
I		44	21	**0.014**
II		16	24	
III–IV		22	23	

The χ^2^ test was used to analyze the relationship between TFAM expression and clinicopathological characteristics. Statistical significance was set at *p* < 0.05.

### Survival analysis

The prognostic value of TFAM for overall survival in NSCLC patients was evaluated by comparing patients with high and low TFAM expression. Kaplan-Meier survival analysis showed that patients with high TFAM expression had distinctly lower overall survival rates than those with low TFAM expression (Figure 5E; *p* = 0.0008). Univariate and multivariate analyses were conducted using Cox's proportional hazards regression model to examine the impact of TFAM expression and other clinicopathological features in NSCLC patients. We found that TFAM expression, age, smoking status, tumor pathological grade, T stage and TNM stage were significant prognostic factors in the univariate analysis (Table 2). We further performed multivariate survival analysis by Cox's proportional hazards regression model to evaluate the effects of independent factors on survival. As shown in Table 2, multivariate analysis indicated that TFAM expression and T stage could serve as independent prognostic factors.

**Table 2:** Univariate analysis and multivariate analysis identifies factors influencing the overall survival of NSCLC patients

Variables	Univariate analysis			Multivariate analysis		
	RR^b^	95%	*p* value	RR^b^	95%	*p* value
**TFAM**	2.845	1.610–5.027	**< 0.001**	2.272	1.157–4.464	**0.017**
**Age**	1.134	1.053–1.221	**0.001**	1.053	0.966–1.148	0.237
**Gender**	1.694	0.938–3.061	0.081	1.631	0.638–4.170	0.308
**Smoking**	1.745	1.022–2.979	**0.041**	0.944	0.401–2.227	0.896
**Alcohol**	0.912	0.490–1.699	0.772	0.627	0.317–1.241	0.180
**Grade**	1.971	1.241–3.131	**0.004**	1.197	0.719–1.933	0.490
**T stage**	7.009	3.781–12.933	**< 0.001**	6.295	2.986–13.269	**< 0.001**
**Lymphnodemetastases**	1.719	1.020–2.896	**0.042**	0.988	0.541–1.844	0.996
**TNM stage**	2.061	1.492–2.847	**< 0.001**	1.053	0.966–1.148	0.063

a Cox's proportional hazards regression model was used to identify the factors that had a significant influence on survival. Statistical significance was set at *p* < 0.05.

b RR, relative risk;

## DISCUSSION

Mitochondria oxidative phosphorylation generates approximately 90% of the ATP in almost all cell types. However, OXPHOS is also the main source of cellular ROS, which may result in mitochondrial dysfunction by oxidation of mtDNA, RNA, proteins, and lipids. TFAM is essential for mtDNA replication and transcription, as well as mtDNA maintenance [7–11]. The TFAM-to-mtDNA ratio is critical for both mtDNA biogenesis and homeostasis, and even small variations in the TFAM-to-mtDNA ratio may affect mitochondrial gene transcription and mtDNA replication [14, 15]. A low TFAM:mtDNA ratio leads to instability of mtDNA and an extremely high TFAM:mtDNA ratio results in the block of mtDNA replication and transcription [14, 15]. HeLa ρ^0^ cells, which are depleted in mtDNA, lose the capacity to form tumors when injected subcutaneously in nude mice [16]; however, the tumorigenicity of HeLa ρ^0^ cells was restored after normal human fibroblast mtDNA or normal DNA-containing mitochondria were introduced [17]. Dong *et al.*, reported that the TFAM mRNA and protein levels were elevated 10- and 11-fold, respectively, in a poorly differentiated rat hepatoma (Morris hepatoma) [18]. TFAM high expression is associated with poor outcome for colorectal cancer patients [13]. TFAM expression levels are elevated in glioma, and positively correlated to the malignancy of glioma [19]. However, the role of TFAM in NSCLC tumorigenesis and migration and the molecular mechanism of its action were undefined.

**Figure 6:**
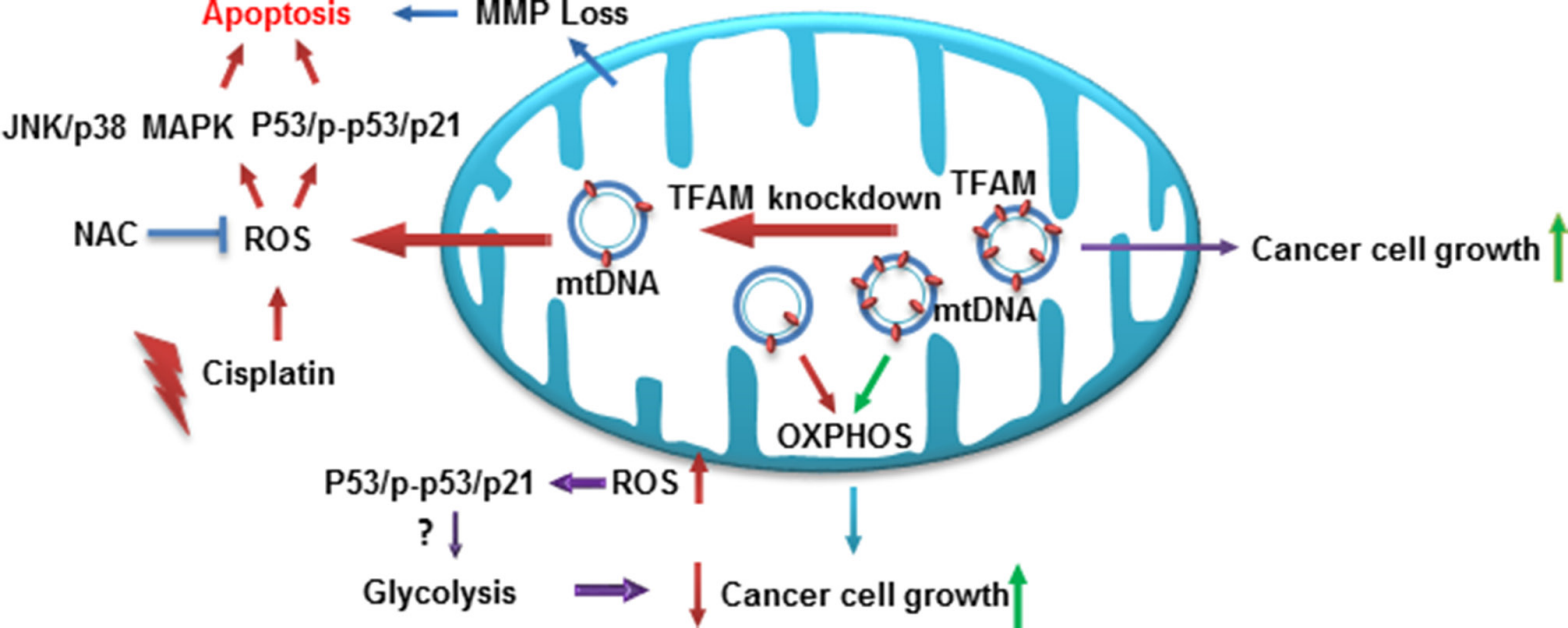
Proposed model of TFAM in modulating JNK/p38 MAPK signaling and cellular bioenergetics TFAM protein expression is critical for NSCLC cancer cell proliferation and tumor growth. Downregulation of TFAM depolarizes the mitochondrial membrane potential, leading to reduced cellular bioenergetics, and increases ROS generation that activates ROS-mediated JNK/p38 MAPK, p53/p-p53 (ser15)/p21 signaling and attenuates cancer cell proliferation and tumor growth.

In the present study, we have shown that downregulation of TFAM inhibits NSCLC tumorigenesis through reducing cellular bioenergetics, activating the ROS-mediated JNK/p38 MAPK and p53/p-p53(ser15)/p21 signaling pathway in NSCLC cells (Figure 6). Our results showed that downregulation of TFAM in NSCLC cells led to inhibition of cell proliferation and colony formation due to cell cycle arrest at G1 phase, as well as increased apoptosis, in both A549 and H460 cells. We also found that TFAM knockdown suppressed tumor growth *in vivo*. In addition, TFAM knockdown significantly decreased NSCLC cell migration. Furthermore, our data showed that both the TFAM mRNA and protein expression levels were significantly increased in NSCLC tumor tissues compared to adjacent normal tissues. mtDNA copy numbers were dramatically increased in the NSCLC tumor tissues compared with tumor adjacent normal tissues, ensuring that cancer cells have sufficient amounts of mtDNA-encoded proteins for remodeling mitochondrial respiratory chain complexes and producing more ATP to support cancer cell proliferation and migration.

At physiological levels, ROS can stimulate cell proliferation and regulate protein function [20, 21]; however, excessive ROS could cause oxidative stress and DNA damage to promote the intrinsic apoptotic pathway [22–24]. Cancer cells generate higher levels of ROS than normal cells [25–27], which is important for cancer cell differentiation. The endogenous antioxidant systems of cancer cells are already stretched to the limits, and even a relatively small increase of ROS disturbs the redox status of cancer cells, which results in cell death and enhances the sensitivity of cancer cells to anti-cancer drugs [28]. p38 acts in concert with JNK to coordinate responses to environmental stimuli or growth signals. Our data demonstrated that TFAM knockdown increased both the accumulation of cellular and mitochondrial ROS and JNK/p38 MAPK phosphorylation, which indicated that downregulation of TFAM results in the inhibition of NSCLC cell proliferation through ROS-mediated JNK and p38 MAPK activation. To determine whether the activation of JNK/p38 MAPK signaling by TFAM knockdown in NSCLC cells was due to ROS, TFAM-knockdown NSCLC and control cells were treated with the ROS scavenger NAC to measure both cellular and mitochondrial ROS levels. We observed that NAC treatment resulted in reduction of ROS and a markedly reduced phospho-p38 protein level. To our surprise, phospho-JNK protein levels declined only slightly after NAC treatment. This finding implies that the activation of p38 MAPK is much more important and sensitive than JNK to TFAM knockdown in NSCLC cells. To better understand the effects of TFAM downregulation in activating the ROS-mediated JNK/p38 MAPK signaling pathway, further investigations are required to clarify the molecular mechanisms operating.

TFAM depletion also resulted in upregulation of the levels of pro-apoptotic Bax, p21, tumor suppressor p53, and p-p53 (ser15), as well as an increase of PARP, caspase 3 and caspase 9 cleavage; however, the expression of anti-apoptotic protein Bcl-2 remained unchanged. p53 upregulation and phosphorylation on serine 15 activated p21, which resulted in cell cycle arrest and the induction of apoptosis. It remains to be elucidated whether p53 upregulation and phosphorylation on serine 15 led to increased cellular and mitochondrial ROS generation in TFAM knockdown NSCLC cells, thereby promoting apoptosis.

Anti-cancer drug resistance contributes to the failure of chemotherapy and relapse, and is still the most important problem and challenge in cancer treatment. Our results showed that knockdown of TFAM enhanced the sensitivity of A549 and H460 NSCLC cells to cisplatin by facilitating ROS-mediated caspase-dependent apoptosis. Thus, our findings indicate that TFAM may play an important role in NSCLC progression via involvement in chemoresistance. Based on our findings, we speculate that downregulation of TFAM may be a potential strategy in NSCLC treatment.

Maintenance of energy homeostasis is critical for tumor cell proliferation, survival, and differentiation. Thus, in many cancer cells, metabolic reprogramming is involved in the switch from OXPHOS to aerobic glycolysis to meet the extremely high demands for energy in support of rapid and uncontrolled cell growth, which is known as the Warburg effect. In this study, we found that knockdown of endogenous TFAM dramatically inhibited OCR and ATP production, which could be attributed to dysfunction in regulating the replication, transcription and maintenance of mtDNA. Interestingly, our data demonstrated that TFAM deficiency also significantly suppressed the cellular glycolytic rate. We have shown that TFAM knockdown in NSCLC cells increased p53 expression and phosphorylation on serine 15, as well as p21 expression. Bensaad and colleagues reported that p53 may activate TIGAR (an inhibitor of the fructose-2, 6-bisphosphate) to negatively regulate glycolysis [29]. Therefore, we proposed that alteration of glycolysis in TFAM deficient cells is partially due to triggering mitochondrial ROS retrograde signaling to promote p53 expression and subsequently repress cellular glycolytic capacity. The reduction in mitochondrial respiration and cellular glycolysis leads to the shortage of energy for NSCLC rapid proliferation and thus inhibits NSCLC tumorigenesis. Moreover, the reduction of OCR in TFAM-knockdown NSCLC cells may also contribute to the increase of ROS, since electon transport chain inhibition is a well-known mechanism of ROS generation [30, 31]. Further studies are warranted to elucidate the molecular mechanism by which TFAM regulates cellular bioenergetics, especially in regulating glycolysis.

To further investigate whether TFAM expression is associated with the progression of NSCLC, the TFAM protein expression levels and the clinic pathologic characteristics of 150 NSCLC patients were analyzed by IHC. Our data indicate that high TFAM protein expression is significantly correlated with tumor grade and TNM stage. Kaplan–Meier survival analysis showed that high TFAM expression is negatively correlated with the overall survival of NSCLC patients. More importantly, further analysis using the Cox's proportional hazards regression model showed that the TFAM expression was an independent factor in predicting overall survival for NSCLC patients. These results suggest that TFAM expression levels might serve as a valuable prognostic biomarker for NSCLC patients after surgery and as a potential therapeutic target in the treatment of NSCLC. Our findings show for the first time that the expression level of TFAM was upregulated in NSCLC cancerous tissues, and reveal an essential role for TFAM protein in NSCLC tumorigenesis.

In conclusion, our study indicates that TFAM supports NSCLC tumorigenesis and may be a novel drug target and prognostic biomarker for NSCLC. These findings also provide a rationale for elucidating the role of TFAM in other types of cancer and for further attempts to block cancer cell proliferation and increase chemosensitivity by downregulating TFAM expression. Finally, TFAM may also be a potential molecular biomarker for preoperative chemotherapy treatment as well as for prediction of the effectiveness of chemotherapy.

## MATERIALS AND METHODS

### Reagents and antibodies

Cisplatin and glucose were obtained from Sigma (St. Louis, MO). Intact cellular oxygen consumption rate (OCR) and extracellular acidification rate (ECAR) assay kits were purchased from Seahorse Bioscience Company (North Billerica, MA). Horseradish peroxidase (HRP)-conjugated anti-rabbit, anti-mouse immunoglobulin G, reactive oxygen species assay kit (DCFH-DA), 5, 5′, 6, 6′-tetrachloro-1, 1′, 3, 3′-tetraethylbenzimidazolylcarbocyanine iodide (JC-1), Bradford protein assay kit and Cell Counting Kit-8 (CCK-8) were obtained from Beyotime (Shanghai, China). Giemsa and crystal violet were purchased from Solarbio Bioscience & Technology (Shanghai, China). Trypan blue was obtained from Life Technologies (Carlsbad, CA). BCA Protein Assay Kit and Pierce ECL Western Blotting Substrate were obtained from Thermo Scientific (Waltham, MA). Polyclonal antibody against TFAM was purchased from Abcam (Cambridge, UK). Monoclonal antibody against β-Actin was from Abmart (Shanghai, China). Antibodies recognizing PARP, Bax, Bcl-2, caspase 3, cleaved caspase 3, caspase 9, cleaved caspase 9, JNK, phospho-JNK (Thr183/Tyr185), p21, p53, p38 and phospho-p38 (Thr180/Tyr182) were obtained from Cell Signaling Technology (Beverly, MA). Phospho-p53 (ser15) antibody was from Beyotime (Shanghai, China). Streptavidin-biotin-peroxidase complex (SABC) and 3, 3′-Diaminobenzidine tetrahydrochloride (DAB) were purchased from Boster (Wuhan, China). Protease (Complete Mini), and phosphatase (PhosphoSTOP) inhibitor cocktail tablets were purchased from Roche Applied Science (Indianapolis, IN).

### Cell lines and cell culture

The human NSCLC cell lines A549, H460 and low-passage HEK293T cells were obtained from the Cell Bank of the Chinese Academy of Sciences (Shanghai, China). A549 and H460 cells were cultured in RPMI-1640 medium (Life Technologies, Grand Island, NY). HEK293T cells were cultured in Dulbecco's Modified Eagle's Medium (DMEM, Life Technologies, Grand Island, NY). All media were supplemented with 10% fetal bovine serum (FBS, Life Technologies, Grand Island, NY) and antibiotics (100 units/ml penicillin and 100 μg/ml streptomycin) at 37°C in a humidified incubator with 5% CO_2_. All cell lines were confirmed mycoplasma free and authenticated by the Cell Bank of the Chinese Academy of Sciences before sending to our lab.

### Patients and samples

NSCLC tissue specimens from both tumor and adjacent normal tissues were obtained from untreated patients who underwent surgical treatment for NSCLC at the Department of Cardiothoracic Surgery, The First Affiliated Hospital of Wenzhou Medical University, Wenzhou, China between January 2006 and March 2015. All samples were cut into two pieces. One piece was embedded in paraffin and processed for routine histopathological examination, while the other piece of tissue was frozen immediately in liquid nitrogen and stored at −80°C for further studies. All patients were clinically and pathologically diagnosed to have NSCLC. Tumor stage was defined according to Union Internationale Contre le Cancer (UICC) and American Joint Committee on Cancer (AJCC) criteria. Tumor pathological grade was defined based on WHO classification.

This study was approved by the Board and Ethical Committee of the First Affiliated Hospital of Wenzhou Medical University, and all patients who participated in this study provided written informed consents in accordance with the Declaration of Helsinki.

### RNA interference

Hairpin-pLKO.1 vectors (carrying a puromycin antibiotic resistance gene) containing control and TFAM-shRNA oligonucleotides were purchased from Biogot Technology (Nanjing, China). The two TFAM shRNAs sequences were as follows: shTFAM#1 5′CCGGGGCAAGTTGTCCAAAGAAACCCTCGAG GGTTTCTTTGGACAACTTGCCTTTTTG3′ (forward); 3′AATTCAAAAAGGCAAGTTGTCCAAAGAAACC CTCGAGGGTTTCTTTGGACAACTTGCC5′ (reverse); shTFAM#2 5′CCGGGGGAACTTCCTGATTCAAAGAC TCGAG TCTTTGAATCAGGAAGTTCCCTTTTTG3′ (forward); 3′AATTCAAAAAGGGAACTTCCTGATTCA AAGACTCGAGTCTTTAATCAGGAAGTTCCC5′ (reverse).

### Lentivirus production and transduction

Lentiviral production and transduction were conducted as previously described [32] and the manufacturer's instructions (GeneCopoeia, Rockville, MD). Briefly, lentiviral vectors and TFAM-shRNA or control shRNA were packed with the Lenti-Pac^™^ HIV Expression Packaging Kit using HEK293T cells and incubated overnight, followed by replacement of the old culture medium with fresh DMEM supplemented with 5% heat-inactivated FBS and penicillin-streptomycin. TiterBoost reagent (1/500 volume) was added to the culture medium and incubated at 37°C in a humidified incubator with 5% CO_2_. At 48 hr post transfection, the supernatants containing lentiviral particles were collected, filtered through 0.45 μm syringe filters (Millipore, Billerica, MA), and used immediately to infect A549 and H460 cells. To select stably transduced cells, the old medium was replaced by fresh complete medium containing puromycin (1 μg/ml) every 3 days until drug-resistant colonies become visible. TFAM knockdown was validated by quantitative real-time PCR and Western blot analysis. Positive clones with stable TFAM knockdown were expanded and maintained in medium supplemented with 1 μg/ml puromycin.

### Cell proliferation assay

A549 and H460 cells with stable TFAM knockdown or vector control cells were seeded into a 24-well plate at a density of 2 × 10^4^ cells per well and incubated overnight. The viability of A549 and H460 cells was determined by counting living and dead cells by the Trypan blue dye (0.05% solution) exclusion method using a hemocytometer.

### Colony formation assay

TFAM stable knockdown A549 and H460 or vector control cells were counted, and a total of 150 cells per well were seeded evenly into 6-well plates and incubated at 37°C for 10–14 days in a humidified incubator with 5% CO_2_. Cells were washed with pre-warmed PBS three times, fixed with 100% methanol and stained with Giemsa solution for 15 min. Colonies were counted by two independent investigators. Each experiment was assayed in triplicate and three independent experiments were performed.

### Transwell cell migration assay

The transwell cell migration assay for TFAM knockdown A549 and H460 or vector control cells was performed as described previously with slight modification [33]. Briefly, cell migration was assayed in transwell cell culture chambers with 6.5 μm diameter polycarbonate membrane filters containing 8 μm pore size (Corning, Tewksbury, MA). A total of 2 × 10^4^ cells in 200 μl of serum-free medium were added to the upper chamber, and the lower chamber was filled with 600 μl culture medium supplemented with 10% FBS. After 48 hr of incubation at 37°C in 5% CO_2_, the non-migrated cells were removed from the upper surface of the membrane with a cotton swab. The migrated cells on the bottom surface were washed with PBS three times, fixed with 4% paraformaldehyde solution, and stained with 0.1% crystal violet. Migrated cells were counted in at least five random microscopic fields (× 100) from each well by two independent investigators. Data are presented as means of three independent experiments performed in triplicate.

### Western blot analysis

Tissue samples were washed 3 times with ice-cold PBS and homogenized using a homogenizer (Kinematica AG, Luzern, Switzerland) in 1.5 ml tissue RIPA lysis buffer (50 mM Tris-HCl, pH 7.4, 1.0% Triton X-100, 1% sodium deoxycholate, 0.1% SDS, 150 mM NaCl) supplemented with protease inhibitor cocktail tablet, NaF (1 mM) and Na_3_VO_4_ (1 mM). Tissue homogenates were cleared by centrifugation at 13,000 rpm for 25 min at 4°C, and the supernatants were collected in clean microcentrifuge tubes on ice. A similar procedure was used to prepare whole cell extracts from cells. Briefly, cells were washed with ice-cold PBS and lysed in RIPA lysis buffer supplemented with protease and phosphatase inhibitors on ice for 20 min, followed by centrifugation at 13,000 rpm for 30 min at 4°C, and the supernatants were collected. Protein concentrations of the tissue homogenates or whole cell extracts were determined using the Pierce BCA protein assay kit.

Tissue or cell extracts equivalent to 20 μg total protein were resolved in 10% SDS-PAGE gels followed by electrophoretic transfer onto nitrocellulose membrane (Bio-Rad, Hercules, CA) in Tris-glycine buffer. Blots were blocked at room temperature for 1.5 hr in 5% non-fat milk in Tris-buffered saline (TBS)-Tween (TBS-T) on a shaker, and then incubated with the primary antibodies indicated in 5% non-fat milk TBS-T overnight at 4°C. The membrane was washed in TBS-T for at least 3 × 10 min and then incubated with horseradish peroxidase (HRP)-conjugated anti-rabbit or anti-mouse immunoglobulin G at room temperature for 1 hr with gentle shaking. Immunreactive proteins were detected by ECL reagent according to the manufacturer's protocol (Thermo Scientific, Rockford, IL). Optical density was measured using National Institute of Health Image J software.

### RNA preparation and quantitative real-time PCR

Total RNAs were extracted from NSCLC tissues or cells using Trizol reagent (Life Technologies, Carlsbad, CA) according to the manufacturer's instructions. Total RNA (2 μg) was used to synthesize first-strand cDNA by reverse transcription using the PrimeScript^™^ RT reagent Kit with gDNA Eraser (Takara, Dalian, China) according to the manufacturer's instructions. The cDNA produced was subsequently used as a template for real-time PCR. Real-time quantitative PCR analysis was performed using 2 μl cDNA/20 μl reaction volume on a CFX Connect^™^ Real-Time PCR Detection System (Bio-Rad, Hercules, CA) using SYBR Green according to the manufacturer's protocol. Primer sequences were as follows: TFAM 5′GAGGGAACTTCCTGATTCAAAGA′3 (forward), 3′AGCTTTCCTTTTTAAATGTTTGTCC′5 (reverse) and β-actin5′CCCTGGCACCCAGCAC′3 (forward), 5′GCCGATCCACACGGAG-TAC′3 (reverse). Thermal cycling was performed using the following parameters: 95°C for 10 min, then 45 cycles of denaturation at 95°C for 10 s and extension at 60°C for 30 s. The threshold cycle number (CT) was recorded for each reaction. The CT value of TFAM was normalized to that of β-actin. Each sample was assayed in triplicate and repeated at least three times.

### FACS analysis for ROS, apoptosis, cell cycle and mitochondrial membrane potential

Intracellular ROS levels were measured as described using the fluorescence probe 2′, 7′-dichlorodihydrofluorescein diacetate (DCFH-DA) according to the manufacturer's protocol (Beyotime, Shanghai, China). DCFH-DA is diffused into cells and deacetylated by esterases to nonfluorescent 2′, 7′-dichlorofluorescin DCFH, which is trapped inside the cells and rapidly oxidized by ROS (including H_2_O_2_) to form highly fluorescent 2′, 7′-dichlorofluorescein (DCF) [34–35]. The fluorescence intensity at 530 nm is proportional to the ROS levels within the cell cytosol. Briefly, TFAM stable knockdown A549 and H460 or vector control cells were trypsinized, washed with PBS, incubated with DCFH-DA at a final concentration of 10 μM in DMEM for 30 min at 37°C, then washed three times with PBS. NAC (5 mM) pre-treatment was employed to confirm ROS production increased in TFAM knockdown NSCLC cells. Mitochondrial superoxide levels were detected by MitoSox staining according to the manufacturer's protocol (Life Technologies, Eugene, OR). Briefly, control and TFAM stable knockdown NSCLC A549 and H460 cells were incubated with 5 μM MitoSOX Red for 10 min at 37°C, and subjected to flow cytometry analysis. Data are plotted as median fluorescence intensity (MFI).

For apoptosis analysis, TFAM stable knockdown A549 and H460 or vector control cells were collected and Annexin V-FITC/PI (BD, San Jose, CA) was added, followed by incubation in the dark at room temperature for 20 min. NAC (5 mM) pre-treatment was employed to confirm that the apoptotic cell death rate increased in TFAM knockdown NSCLC cells.

To analyze the cell cycle, TFAM stable knockdown A549 and H460 or vector control cells were seeded in 6-well plates in triplicate and incubated in a 37°C, 5% CO_2_ incubator for 48 hr. Cells were then harvested, washed twice in ice-cold phosphate-buffered saline (PBS), and fixed in 70% ice-cold ethanol overnight at 4°C. On the next day, the cells were washed twice with ice-cold PBS and incubated with propidium iodide (PI, 50 μg/ml) solution for 30 min at 37°C in the dark.

For mitochondrial membrane potential analysis, TFAM stable knockdown A549 and H460 or vector control cells were stained with 2.0 μM JC-1 in complete medium, and incubated for 20 min at 37°C in the dark [36]. To remove excess JC-1, cells were washed once with warm PBS and pelleted by centrifugation. Cell pellets were resuspended by gently flicking the tubes, and 500 μl PBS (room temperature) were added to each tube.

Cell samples were analyzed immediately using a BD Accuri^™^ C6 flow cytometer (BD, Franklin Lakes, NJ).

### Cytotoxic assays and cisplatin-sensitivity assay

To determine the mean inhibitory concentration (IC_50_) for cisplatin, TFAM stable knockdown A549 and H460 cells or the control cells were seeded in 96 well plates at a density of 5 × 10^3^ cells/well and incubated overnight at 37°C, 5% CO_2_. On the next day, cells were treated with cisplatin at a variety of concentrations (1, 2, 4, 8 or 16 μg/ml) or saline solution as the vehicle control. At 24 hr after cisplatin treatment, cells were incubated with CCK-8 for 2 hr. The absorbance at 450 nm was measured using a Varioskan Flash microplate reader (Thermo Scientific, Waltham, MA). Cisplatin stock solution was made freshly 24 hr before each experiment in saline solution medium to minimize the hydrolysis of cisplatin [37]. Each assay was performed in triplicate and data was derived from at least three independent experiments.

### Caspase 3 activity assay

Caspase 3 activity was assayed using the Caspase 3 Activity Assay Kit (Beyotime Shanghai, China) according to the manufacturer's protocol. In brief, 50 μl of cell lysate was mixed in 96-well microtiter plates with 10 μl Ac-DEVD-pNA (2 mM) in 100 μl reaction buffer, incubated at 37°C for 2 hr, and the absorbance measured at 405 nm using a Varioskan Flash microplate reader (Thermo Scientific, Waltham, MA). Once the assay was completed, a Bradford Protein Assay Kit (Beyotime Haimen, China) was used to determine the protein concentration to normalize the relative caspase 3 activity.

### Oxidative phosphorylation and glycolysis assay

The intact cellular oxygen consumption rate (OCR) and extracellular acidification rate (ECAR) of TFAM knockdown A549 and H460 and control cells was measured using a Seahorse XF-96 Extracellular Flux Analyzer (Seahorse Bioscience, North Billerica, MA) as described [38]. Results were obtained by performing three independent experiments with 8 replicates of control and TFAM knockdown A549 and H460 cells include. After the assay was completed, a BCA Protein Assay Kit was used according to the manufacturer's instructions to determine the protein concentration to normalize OCR and ECAR.

### Tissue microarrays (TMA) and immunohistochemical (IHC) staining

A total of 150 formalin-fixed, paraffin-embedded NSCLC tissues were prepared for construction of the TMA as described by Nocito *et al.* [39]. IHC staining was performed following previously described protocols with minor modifications [38]. The TMA slides were incubated with the primary antibodies against TFAM or PBS as a negative control at 4°C overnight, followed by incubation with biotin-labeled goat anti-rabbit IgG and then with streptavidin-biotin-peroxidase complex (SABC). IHC staining was visualized by use of 3, 3′-Diaminobenzidine tetrahydrochloride (DAB), and counterstaining was with hematoxylin.

### Evaluation of IHC staining

All TMA samples were viewed and photographed under a Nikon Light Microscope. IHC images were reviewed and scored independently by two pathologists who had no prior knowledge of the clinicopathological features of the specimens on TMA. The relative expression of TFAM was evaluated according to the protocol we previously described [38].

### Xenograft tumor assay in nude mice

Female athymic nude mice were purchased from Shanghai Laboratory Animal Center, CAS (Shanghai, China) and housed in a specific pathogen-free (SPF) environment. For *in vivo* tumorigenesis analysis, nude mice at the age of 5 weeks were injected subcutaneously in the left and right flanks with 6 × 10^6^ of vector control or TFAM-knockdown H460 cells in 100 μml of serum-free PBS. The mice were sacrificed 23 days after injection and the tumors were harvested and weighted. Tumors tissues were homogenized and lysed for western blotting analysis. All animal studies were performed with an approved protocol by the Institutional Animal Care and Use Committee of Wenzhou Medical University.

### Statistical analysis

The expression level of TFAM was quantified relative to β-actin, a loading control protein. All statistical analyses were performed with the SPSS 16.0 statistical software package (SPSS Standard version 16.0, SPSS Inc., Chicago, IL). The Wilcoxon signed-rank test was used to analyze the significance of protein and mRNA expression in adjacent normal and cancer tissues of NSCLC. The χ^2^ test was performed to evaluate the relationship between the clinic pathological features and TFAM expression in the IHC results. Kaplan–Meier survival analysis was used to evaluate patients' prognoses. The Cox's proportional hazards regression model was performed to identify factors influencing the overall survival of NSCLC; *p* ≤ 0.05 was considered to be statistically significant. Data are shown as means ± SD.

## SUPPLEMENTARY MATERIALS FIGURES


