# Supplementary material for: Downregulation of TFAM inhibits the tumorigenesis of non-small cell lung cancer by activating ROS-mediated JNK/p38MAPK signaling and reducing cellular bioenergetics
Source: Oncotarget. 2016 Jan 25;7(10):11609–24. doi: 10.18632/oncotarget.7018 (PMC4905497; doi:10.18632/oncotarget.7018)
Supplement: Supplementary file 1 [file oncotarget-07-11609-s001.pdf]

# Downregulation of TFAM inhibits the tumorigenesis of non-small cell lung cancer by activating ROS-mediated JNK/p38MAPK signaling and reducing cellular bioenergetics

## Supplementary Materials

### MtDNA copy number assay

Total genomic DNA was extracted from TFAM stable knockdown A549 and H460 cells or from NSCLC tumor tissues (C) and adjacent normal tissues (N) by TaKaRa MiniBEST Universal Genomic DNA Extraction Kit Ver.5.0 (Takara, Dalian, China). DNA was quantified and Real-time quantitative PCR (qRT-PCR) was performed to analyze the relative mtDNA copy numbers in both cells and tissues samples. Thermal cycling was performed using the following parameters: 95°C for 10 min, 40 cycles at 95°C for 5 sec and 58°C for 30 sec. All reactions were performed in triplicate with identical amounts of input genomic DNA, and relative amplifications of the nuclear DNA (nDNA) encoded gene *β-ACTIN* and the *ND1* sequences of mtDNA were determined using the comparative ΔΔCt method. The data represent at least three independent experiments and the standard error of the mean is indicated. Sequences of primers as follows: *β-ACTIN* forward: 5′-TCCCAGCACACTTAACTTAGC-3′; reverse: 5′-AGC CACAAGAAACACTCAGG-3′ and *ND1* forward: 5′-CAC CCAAGAACAGGGTTTGT-3′; reverse: 5′-TGGCCATG GGATTGTTGTTAA-3′.

### Transfection of TFAM siRNA

A549 and H460 cells were transfected with control or TFAM siRNA (GenePharma, Shanghai, China) using Lipofectamine 3000 (Life Technologies, Grand Island, NY) for 24, 48 and 72 hr in serum-free medium following manufacturer's instructions. The following sequences for TFAM siRNA are: sense 5′-GGCAAGUUGUCCAAAGAAATT-3′; antisense 5′-U UUCUUUGGACAACUUGCCTT-3′ and the sequences for control siRNA are: sense 5′-UUCUCCGAACGUG UCACGUTT-3′; antisense 5′-ACGUGACACGUUCGG AGAATT- 3′.

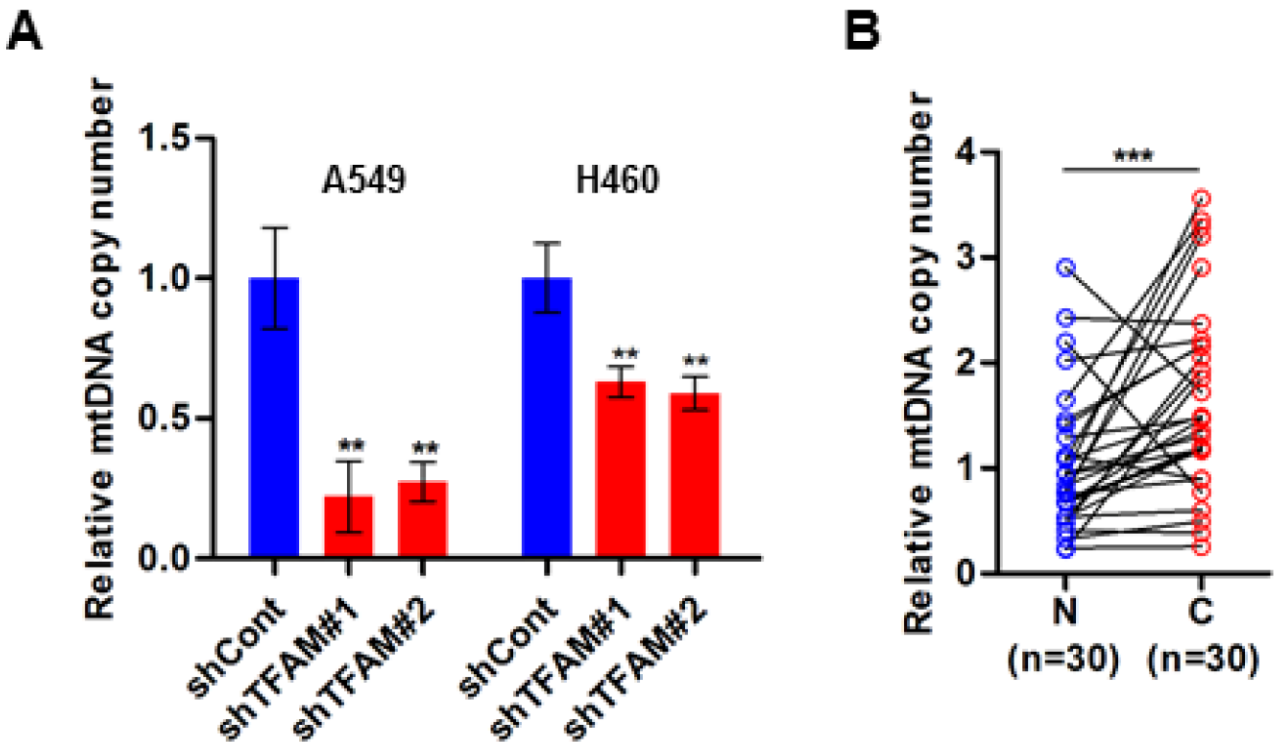


**Supplementary Figure S1: Relative mtDNA copy numbers.** (**A**) mtDNA copy numbers in TFAM stable knockdown A549 and H460 cells. (**B**) mtDNA copy numbers in NSCLC tumor tissue (C) and adjacent normal tissue (N) were examined by qRT-PCR and quantitated using the comparative $\Delta\Delta C_t$ method. The data are presented as mean ± SD (A, $n = 3$, $^{**}P < 0.005$; B, $n = 30$, $^{***}P < 0.001$).

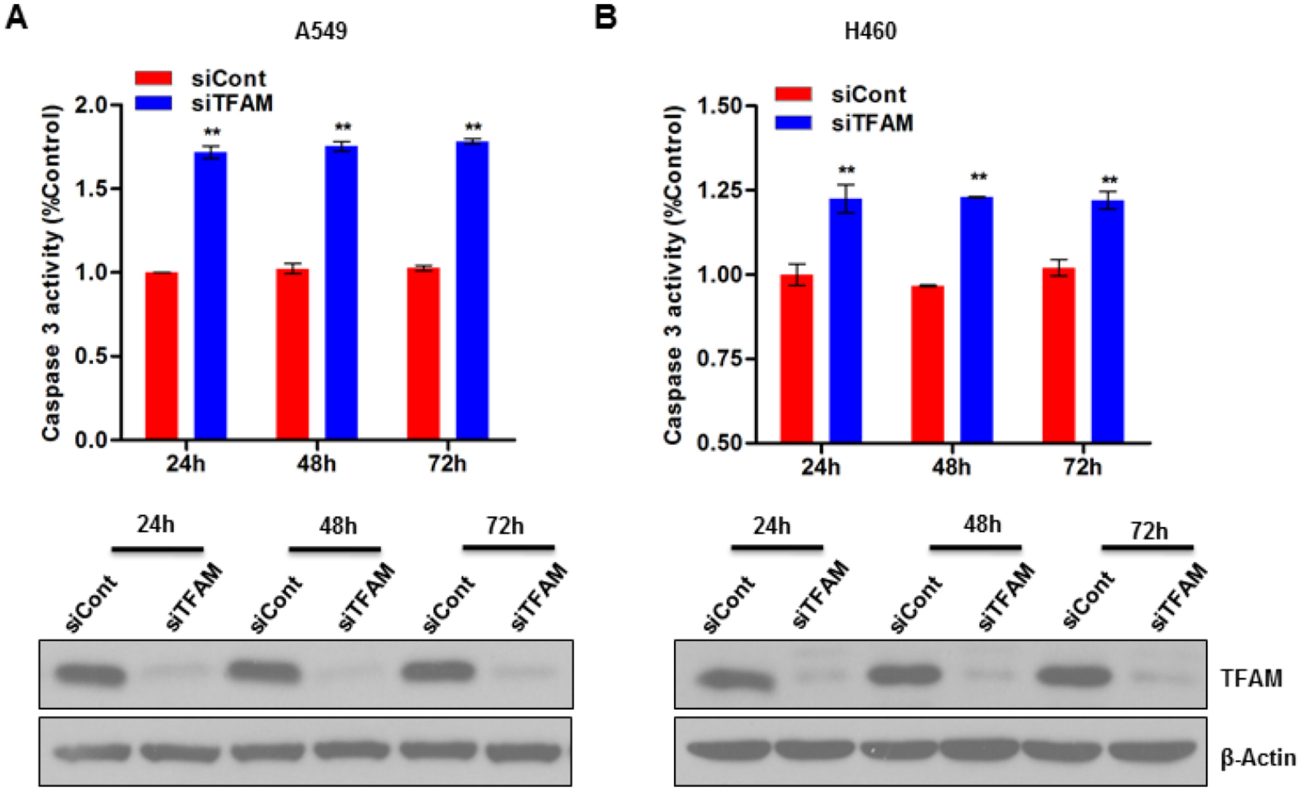


**Supplementary Figure S2: Caspase 3 activity in TFAM knockdown NSCLC cells is stable over time.** NSCLC A549 (**A**) and H460 (**B**) cells were transfected with control (siCont) or TFAM siRNA (siTFAM). Cells were collected at 24, 48 and 72 hr post transfection and caspase 3 activity was assayed using the Caspase 3 Activity Assay kit. The data are presented as mean ± SD (upper panel; $n = 3$, $^{*}p < 0.05$; $^{**}p < 0.01$). TFAM knockdown was confirmed by western blot (bottom panel).
